# Higher Loading Dose of Colistin for the Treatment of Ventilator-Associated Pneumonia in Critically Ill Children: A Retrospective Comparative Study at Queen Rania Abdullah Hospital for Children

**DOI:** 10.7759/cureus.115828

**Published:** 2026-09-05

**Authors:** Shadi H Shweiyat, Nasser Banikhaled, Yacoub M Haddadin, Hala Alhajaj, Shaden Alsaryrah, Amjed Abulannaz, Anees Hjazeen, Adnan Aldwan

**Affiliations:** 1 Pediatric Critical Care, Jordanian Royal Medical Services, Amman, JOR; 2 Pediatric Infectious Diseases, Jordanian Royal Medical Services, Amman, JOR; 3 Pediatric Intensive Care Unit, Queen Rania Al Abdullah Hospital for Children, Amman, JOR; 4 Pediatric Critical Care, Queen Rania Al Abdullah Hospital for Children, Amman, JOR; 5 Microbiology, Jordanian Royal Medical Services, Amman, JOR; 6 Pediatrics, Jordanian Royal Medical Services, Amman, JOR; 7 Community Health Nursing/Biostatistics, Jordanian Royal Medical Services, Amman, JOR

**Keywords:** carbapenem-resistant gram-negative infection, colistin resistance, loading dose, pediatric intensive care unit (picu), ventilator-associated pneumonia

## Abstract

Background: Ventilator-associated pneumonia (VAP) remains a significant cause of morbidity and mortality in pediatric intensive care units (PICUs), particularly when caused by multidrug-resistant (MDR) Gram-negative organisms. Colistin has re-emerged as a last-line antibiotic for these infections; however, optimal dosing strategies remain inadequately studied in children.

Objective: This retrospective study evaluates the association between a higher loading dose of colistin (9 mg/kg colistin base activity (CBA)) compared to the standard loading dose (5 mg/kg CBA) and clinical outcomes in children with VAP caused by carbapenem-resistant organisms.

Methods: A retrospective comparative study was conducted at the PICU, Queen Rania Abdullah Hospital for Children, Royal Medical Services, Amman, Jordan, covering a three-year period from January 2023 to January 2026. Medical records of 80 children aged one month to 14 years with confirmed VAP caused by colistin-susceptible MDR Gram-negative organisms were reviewed. Patients were categorized according to colistin loading dose into group A, which received a standard loading dose of 5 mg/kg CBA (n = 40), and group B, which received a higher loading dose of 9 mg/kg CBA (n = 40). Both groups received a maintenance dose of 2.5 mg/kg CBA every 12 hours.

Results: Among 80 children (mean age = 4.8 ± 3.6 years, range = 1 month to 12 years; 45/80 (56.3%) male), the higher loading dose group demonstrated significantly higher microbiological eradication at day 7 (33/40 (82.5%) vs. 24/40 (60.0%), p = 0.028). The clinical cure rate was also higher in the higher dose group (31/40 (77.5%) vs. 22/40 (55.0%), p = 0.034). Median time to clinical response was shorter in the higher dose group (4 vs. 6 days, p = 0.018). Colistin trough levels on day 2 were significantly higher in the higher dose group (2.8 ± 0.9 vs. 1.6 ± 0.6 µg/mL, p < 0.001), with a higher proportion achieving therapeutic levels (≥2 µg/mL) (29/40 (72.5%) vs. 14/40 (35.0%), p = 0.001). Duration of mechanical ventilation was shorter in the higher dose group (median = 12 vs. 16 days, p = 0.042). PICU length of stay was also reduced (median = 18 vs. 24 days, p = 0.038). Nephrotoxicity incidence was similar between groups (9/40 (22.5%) vs. 7/40 (17.5%), p = 0.58). In multivariate analysis, a higher loading dose was independently associated with microbiological eradication (adjusted odds ratio (aOR) = 3.24, 95% CI: 1.28-8.20, p = 0.013) and clinical cure (aOR = 2.86, 95% CI: 1.12-7.30, p = 0.028).

Conclusions: A higher loading dose of colistin (9 mg/kg CBA) in children with VAP caused by carbapenem-resistant organisms was associated with improved microbiological eradication, faster clinical response, and shorter duration of mechanical ventilation and PICU stay, while nephrotoxicity did not differ significantly between groups. These findings are hypothesis-generating and require confirmation in larger, prospective, preferably multicenter randomized studies.

## Introduction

Ventilator-associated pneumonia (VAP) remains one of the most serious healthcare-associated infections in pediatric intensive care units (PICUs), contributing significantly to morbidity, mortality, and healthcare resource utilization [1,2]. The incidence of VAP in critically ill children ranges from two to 20 per 1000 ventilator days, with mortality rates reported as high as 20-30% in affected patients [3,4]. The emergence of multidrug-resistant (MDR) and extensively drug-resistant (XDR) Gram-negative organisms, particularly carbapenem-resistant *Acinetobacter baumannii*, *Pseudomonas aeruginosa*, and *Klebsiella pneumoniae*, has substantially complicated the management of VAP in pediatric populations [5,6].

Colistin (polymyxin E) has re-emerged as a last-line antibiotic for the treatment of infections caused by MDR Gram-negative organisms, following the decline in the development of new antibiotics active against these pathogens [7,8]. Despite its efficacy, colistin use is limited by its narrow therapeutic window and significant nephrotoxicity, which occurs in up to 30-50% of treated patients [9,10]. Additionally, colistin exhibits concentration-dependent killing and requires adequate exposure to achieve clinical efficacy [11,12].

Pharmacokinetic studies have demonstrated that colistin requires a loading dose to rapidly achieve therapeutic plasma concentrations, given its slow distribution and prolonged time to steady state with maintenance doses alone [13,14]. Current international guidelines recommend a loading dose of 5 mg/kg of colistin base activity (CBA), followed by a maintenance dose of 2.5 mg/kg CBA every 12 hours for critically ill patients, including children [14,15]. However, recent evidence suggests that higher loading doses (9 mg/kg CBA) may achieve more rapid therapeutic concentrations and improve clinical outcomes without increasing nephrotoxicity [16,17].

Data on optimal colistin dosing in children have been growing, with several studies demonstrating the pharmacokinetic and clinical benefits of loading doses. Antachopoulos et al. (2021) demonstrated that higher than recommended doses of colistin were associated with improved clinical outcomes in critically ill pediatric patients, with population pharmacokinetic analysis supporting the safety and efficacy of this approach [18]. Lu et al. (2012) demonstrated that high-dose nebulized colistin was more effective in VAP caused by MDR *Pseudomonas aeruginosa* and *Acinetobacter baumannii* [19]. Fatehi et al. (2019) demonstrated significantly higher overall efficacy with a colistin loading dose (150,000 IU/kg, approximately 5 mg/kg CBA) in critically ill children [20]. Garonzik et al. (2011) showed that a colistin loading dose of 4 mg/kg CBA resulted in improved colistin exposure without increased acute kidney injury [21]. However, the optimal loading dose and its safety profile in different pediatric subpopulations require further investigation.

In Jordan, the prevalence of carbapenem-resistant organisms has been rising, posing a significant challenge to the management of VAP in PICUs [22,23]. At Queen Rania Abdullah Hospital for Children, colistin is frequently used as a last-line agent for MDR Gram-negative VAP. However, the optimal loading dose in this population has not been systematically evaluated.

This study aimed to compare the association of a 9 mg/kg versus 5 mg/kg CBA colistin loading dose with microbiological eradication at day 7 in children with VAP caused by carbapenem-resistant organisms, while secondarily evaluating clinical cure, time to clinical response, colistin trough levels and achievement of therapeutic concentrations, nephrotoxicity, duration of mechanical ventilation, PICU length of stay, and 28-day mortality.

## Materials and methods

Ethics statement

This study received ethical approval from the Institutional Review Board (IRB) of the Jordanian Royal Medical Services (Registration Number: 39_10/2026) on June 20, 2026. Final authorization for data dissemination and publication was provided by the Jordanian Royal Medical Services Educational and Training Directorate on August 18, 2026. The study was conducted in accordance with the ethical principles outlined in the Declaration of Helsinki. The IRB granted a waiver for individual informed consent due to the retrospective, anonymized nature of the research.

Study design and setting

A retrospective comparative study was conducted at the Pediatric Intensive Care Unit, Queen Rania Abdullah Hospital for Children, Royal Medical Services, Amman, Jordan, covering a three-year period from January 2023 to January 2026. The study is reported in accordance with the STROBE guidelines for observational studies [24].

Study population

Medical records of all children aged one month to 14 years with a confirmed diagnosis of VAP caused by colistin-susceptible MDR Gram-negative organisms who received colistin therapy were screened for eligibility. Table 1 presents the inclusion and exclusion criteria for study participation.

**Table 1:** Inclusion and exclusion criteria. Abbreviations: PICU, pediatric intensive care unit; VAP, ventilator-associated pneumonia; MDR, multidrug-resistant; CFU, colony-forming units.

Criterion type	Criteria
Inclusion criteria	
1	Age 1 month to 14 years
2	Admission to PICU with invasive mechanical ventilation for ≥48 hours
3	Confirmed diagnosis of VAP based on positive quantitative culture (endotracheal aspirate ≥10⁵ CFU/mL or bronchoalveolar lavage ≥10⁴ CFU/mL), new or progressive infiltrate on chest radiograph, and at least two clinical signs (fever >38.5°C or hypothermia <35.0°C, leukocytosis >15,000/µL or leukopenia <4,000/µL, or purulent tracheal secretions)
4	Isolation of colistin-susceptible MDR Gram-negative organism
5	Received colistin therapy for ≥72 hours
6	Complete medical records
Exclusion criteria	
1	Pre-existing chronic kidney disease (baseline creatinine >1.5× upper limit of normal for age)
2	Receiving concurrent nephrotoxic agents (other than colistin) such as aminoglycosides or vancomycin
3	Known allergy to colistin
4	Colistin therapy for <72 hours
5	Incomplete medical records

Colistin dosing

During the study period (January 2023 to January 2026), a clinical protocol change occurred in our PICU regarding colistin dosing. From January 2023 to December 2023, the institutional protocol recommended a standard loading dose of 5 mg/kg CBA. From January 2024 onwards, following the publication of emerging evidence supporting higher colistin loading doses and a local multidisciplinary review involving pediatric intensivists, infectious disease specialists, and clinical pharmacists, the protocol was updated to recommend a higher loading dose of 9 mg/kg CBA.

Patients were categorized into two groups based on the colistin loading dose actually administered: group A (standard loading dose), receiving a loading dose of 5 mg/kg CBA (n = 40); and group B (higher loading dose), receiving a loading dose of 9 mg/kg CBA (n = 40).

The final sample resulted in exactly 40 patients per group because we selected the first 40 eligible patients treated under each protocol period to ensure balanced group sizes for optimal statistical comparison.

Both groups received a maintenance dose of 2.5 mg/kg CBA every 12 hours.

Colistin was administered as colistimethate sodium (CMS) for intravenous injection. Doses were expressed as CBA. The conversion used was 1 mg CBA = 30,000 IU CMS. The maximum loading dose was 270 mg CBA (equivalent to 8.1 million IU CMS) for patients weighing ≥30 kg; for patients weighing <30 kg, the maximum dose was capped at 9 mg/kg CBA. Colistin was administered as a 30-minute intravenous infusion using an infusion pump. The first maintenance dose (2.5 mg/kg CBA) was administered 12 hours after completion of the loading dose. For patients with estimated glomerular filtration rate (eGFR) <30 mL/min/1.73m², the maintenance dose interval was extended to 24 hours. For patients with eGFR between 30 and 60 mL/min/1.73 m², the maintenance dose was adjusted to 1.25 mg/kg CBA every 12 hours. The loading dose was not adjusted for renal impairment.

Data collection

Data were extracted from electronic medical records using a standardized case report form. Variables collected included demographic and clinical data, microbiological data, laboratory data, and clinical outcomes. The Pediatric Risk of Mortality (PRISM) III score was calculated for each patient at admission [25].

Endotracheal aspirate specimens were collected using a sterile closed-suction system under aseptic conditions. Quantitative cultures were performed by inoculating serial dilutions onto appropriate agar plates (blood agar, MacConkey agar, and chocolate agar) and incubating at 37°C for 18-24 hours. Specimens were considered positive if growth was ≥10⁵ CFU/mL for endotracheal aspirates or ≥10⁴ CFU/mL for bronchoalveolar lavage. Organism identification was performed using the VITEK 2 automated identification system (bioMérieux, Marcy-l'Étoile, France). Antimicrobial susceptibility testing was performed using the broth microdilution method for colistin and the disk diffusion method for other antibiotics, following Clinical and Laboratory Standards Institute (CLSI) guidelines [26]. Colistin susceptibility was defined as a minimum inhibitory concentration (MIC) ≤2 µg/mL, in accordance with the interpretive standards of the European Committee on Antimicrobial Susceptibility Testing (EUCAST) and the CLSI [26,27]. Carbapenem resistance was confirmed by an MIC ≥4 µg/mL for meropenem or imipenem.

Colistin trough levels were measured immediately before the third maintenance dose (approximately 36 hours after the loading dose), representing the steady-state trough concentration. Blood samples (3 mL) were collected in heparin-free serum separator tubes, and serum was separated and stored at -80°C until analysis. Colistin concentrations were measured using a validated liquid chromatography-tandem mass spectrometry (LC-MS/MS) method with a lower limit of quantification of 0.1 µg/mL. The ≥2 µg/mL therapeutic threshold was selected based on pharmacokinetic/pharmacodynamic targets established in the literature, which suggest that this concentration is associated with improved clinical outcomes and corresponds to the colistin susceptibility breakpoint (MIC ≤2 µg/mL) for most MDR Gram-negative organisms [14,15].

Combination therapy was administered in both groups as per institutional protocol for carbapenem-resistant organisms. Patients in both groups may have received other active antibiotics as part of their treatment regimen for VAP. Combination therapy was administered in 70.0% (28/40) of patients in the standard dose group and 75.0% (30/40) of patients in the higher dose group (p = 0.62). The specific combination agents included carbapenems (meropenem or imipenem), rifampicin, or tigecycline, prescribed at the discretion of the treating physician based on susceptibility results and clinical judgment.

Outcome measures

The primary outcome of this study was microbiological eradication at day 7, defined as negative tracheal culture or a significant reduction in colony count (≥2 log₁₀ reduction) on day 7 culture. Secondary outcomes included the following: clinical cure, defined as resolution of fever, normalization of white blood cell count, and improvement in oxygenation index by day 7; time to clinical response measured in days; colistin trough levels (µg/mL) on day 2 and day 5; the proportion of patients achieving therapeutic trough levels (≥2 µg/mL); duration of mechanical ventilation in days; PICU length of stay in days; 28-day mortality; and nephrotoxicity, defined using the modified Kidney Disease: Improving Global Outcomes (KDIGO) criteria: a ≥50% increase in serum creatinine from baseline, a ≥0.5 mg/dL increase in serum creatinine, or urine output <0.5 mL/kg/hour for ≥6 hours [28]. Severity of nephrotoxicity was further stratified using the KDIGO staging system (Stage 1: creatinine 1.5-1.9× baseline or ≥0.3 mg/dL increase; Stage 2: creatinine 2.0-2.9× baseline; Stage 3: creatinine ≥3.0× baseline or initiation of renal replacement therapy).

Statistical analysis

All statistical analyses were performed using SPSS version 26.0 (IBM Corp., Armonk, NY) and R version 4.2.2 (R Foundation, Vienna, Austria) for graphical outputs. A two-tailed p-value <0.05 was considered statistically significant.

Descriptive Statistics

Continuous variables are presented as means ± standard deviations or medians with interquartile ranges, as appropriate. Categorical variables are presented as frequencies with percentages (n, %).

Univariate Comparisons

Independent t-tests were used for normally distributed continuous variables, Mann-Whitney U tests for non-normally distributed continuous variables, and chi-square tests or Fisher's exact tests for categorical variables, as appropriate. Normality was assessed using the Shapiro-Wilk test and visual inspection of Q-Q plots.

Multivariable Logistic Regression

Binary logistic regression was used to identify independent predictors of clinical cure. Variables were selected for entry into the model based on clinical relevance, biological plausibility, and a univariate screening threshold of p < 0.10. The following variables were entered as covariates: (1) colistin loading dose group (5 mg/kg vs. 9 mg/kg CBA); (2) PRISM III score (categorized as <20 vs. ≥20); (3) organism type (*Acinetobacter baumannii* vs. other) [25]; (4) colistin trough ≥2 µg/mL at day 2 (yes/no); (5) combination therapy (yes/no); (6) baseline eGFR (continuous); and (7) timing of appropriate therapy (hours from VAP diagnosis to initiation of colistin). Stepwise forward selection was used with an entry criterion of p < 0.10 and a removal criterion of p > 0.05. Multicollinearity was assessed using the variance inflation factor (VIF), with values >5 indicating significant collinearity. Model fit was assessed using the Hosmer-Lemeshow goodness-of-fit test (p > 0.05 indicating adequate fit) and the Cox-Snell and Nagelkerke R² statistics.

Missing Data Management

Missing data were minimal across all variables (<2% for demographic and clinical variables). Colistin trough levels were not available for eight patients (four in each group, 10% of total) due to logistical reasons (inability to draw blood at the precise sampling time or sample hemolysis). These patients were excluded from trough-level analyses only but were retained in all other analyses. Complete-case analysis was used for all multivariable models, as the proportion of missing data was below the threshold (<10%) considered to introduce significant bias.

Sample Size Consideration

With 40 patients per group and an estimated clinical cure rate of 55% in the standard group and 77.5% in the higher dose group, the study had 80% power to detect this difference at α = 0.05 (two-tailed), based on a chi-square test for two independent proportions.

## Results

Participant characteristics

Table 2 presents the baseline demographic and clinical characteristics of the 80 children included in the final analysis. A total of 80 children were included in the final analysis: 40 in the standard loading dose group (group A) and 40 in the higher loading dose group (group B). Baseline demographic and clinical characteristics were well-balanced between groups. The mean age was 4.8 ± 3.6 years (range = 1 month to 12 years), with 45/80 (56.3%) male. The most common organisms isolated were *Acinetobacter baumannii* (34/80, 42.5%), *Klebsiella pneumoniae* (25/80, 31.3%), and *Pseudomonas aeruginosa* (21/80, 26.3%). Figure 1 illustrates the participant screening, exclusion, and final cohort allocation process.

**Table 2:** Baseline demographic and clinical characteristics. Abbreviations: CBA, Colistin Base Activity; SD, Standard Deviation; PRISM III, Pediatric Risk of Mortality III.

Characteristic	Standard loading dose (5 mg/kg CBA) (n = 40)	Higher loading dose (9 mg/kg CBA) (n = 40)	p-value
Age (years), Mean ± SD	4.6 ± 3.4	5.0 ± 3.8	0.62
Male, n (%)	22/40 (55.0)	23/40 (57.5)	0.82
PRISM III score, Mean ± SD	18.8 ± 6.4	18.0 ± 6.0	0.56
Acinetobacter baumannii, n (%)	18/40 (45.0)	16/40 (40.0)	0.58
Klebsiella pneumoniae, n (%)	12/40 (30.0)	13/40 (32.5)	0.58
Pseudomonas aeruginosa, n (%)	10/40 (25.0)	11/40 (27.5)	0.58

**Figure 1:**
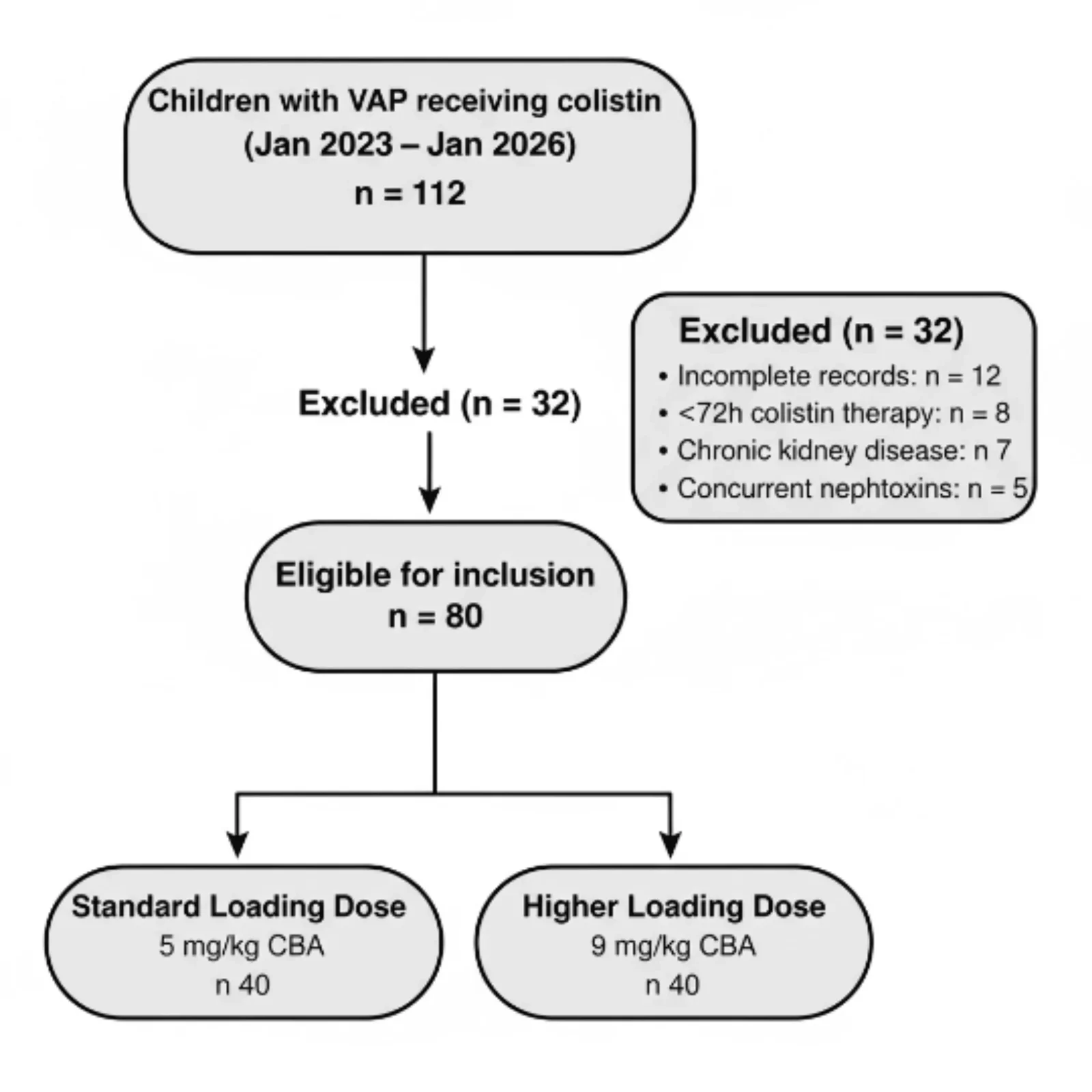
Participant flow diagram. Of the 112 children initially screened for eligibility, 32 were excluded for various reasons, including incomplete medical records (n = 12), colistin therapy for less than 72 hours (n = 8), pre-existing chronic kidney disease (n = 7), and concurrent use of nephrotoxic agents (n = 5). The remaining 80 patients met all inclusion criteria and were stratified into two groups based on the colistin loading dose administered: the standard loading dose group (5 mg/kg CBA, n = 40) and the higher loading dose group (9 mg/kg CBA, n = 40). Abbreviations: VAP, ventilator-associated pneumonia; CBA, colistin base activity; h, hours.

Microbiological eradication

Table 3 presents the microbiological outcomes at day 7 of colistin therapy between the two groups. The higher loading dose group demonstrated significantly higher microbiological eradication at day 7 compared to the standard dose group (33/40 (82.5%) vs. 24/40 (60.0%), χ² = 4.82, df = 1, p = 0.028, Cramér's V = 0.25). Figure 2 presents a bar chart comparing microbiological eradication rates at day 7 between the two treatment groups.

**Table 3:** Microbiological outcomes. Abbreviations: CBA, colistin base activity; IQR, interquartile range.

Outcome	Standard loading dose (5 mg/kg CBA) (n = 40)	Higher loading dose (9 mg/kg CBA) (n = 40)	p-value
Microbiological eradication at day 7, n (%)	24/40 (60.0)	33/40 (82.5)	χ² = 4.82, df = 1, p = 0.028, Cramér's V = 0.25
Time to negative culture (days), median (IQR)	8 (6-14)	5 (4-10)	p = 0.012

**Figure 2:**
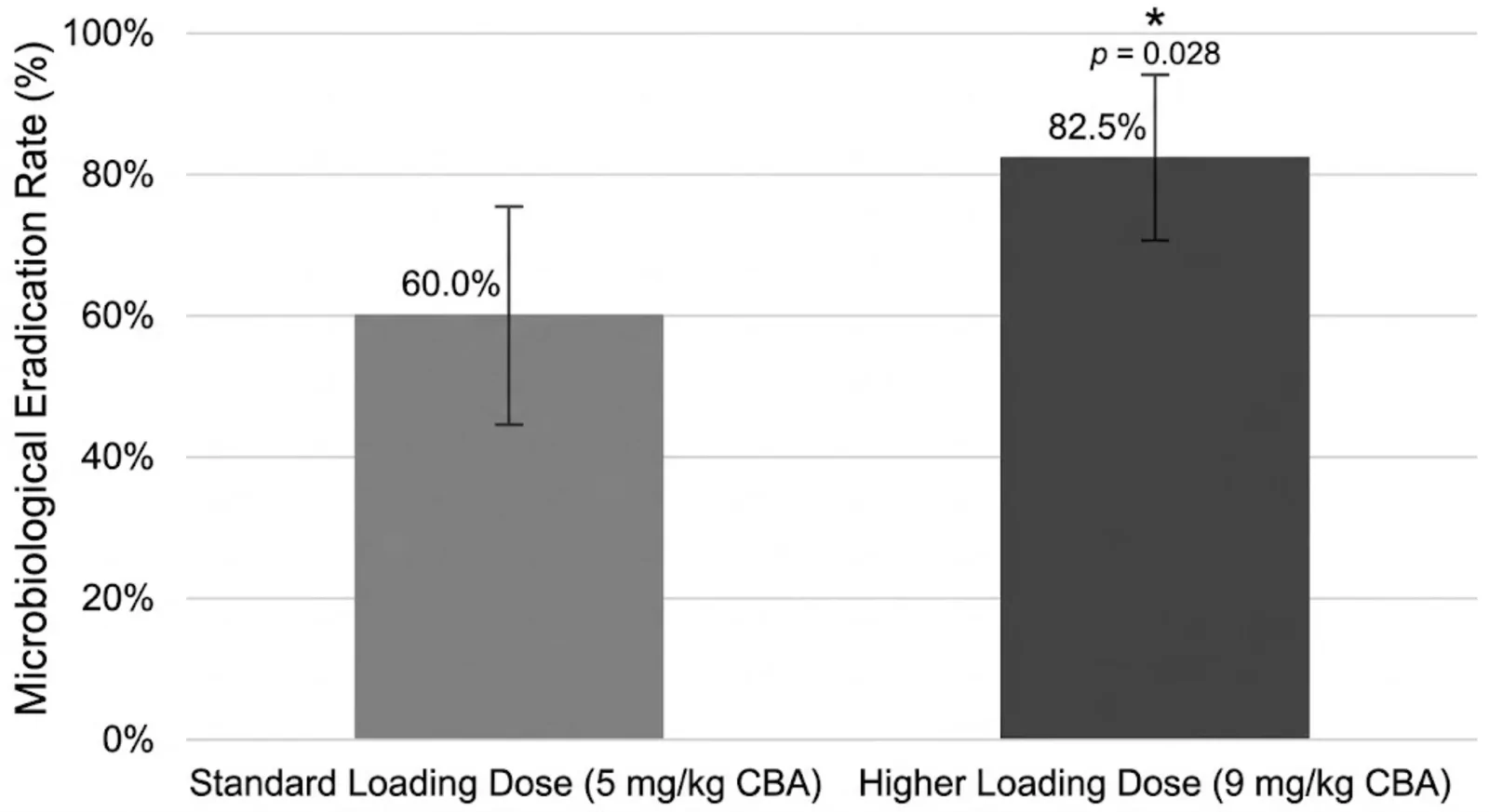
Microbiological eradication at day 7. This bar chart compares microbiological eradication rates at day 7 between the two treatment groups. The higher loading dose group (9 mg/kg CBA) demonstrated significantly higher microbiological eradication rates compared to the standard loading dose group (5 mg/kg CBA), with eradication rates of 33/40 (82.5%) versus 24/40 (60.0%), respectively (p = 0.028). Error bars represent 95% confidence intervals. * indicates statistical significance (p < 0.05). Abbreviations: CBA, colistin base activity; CI, confidence interval.

Clinical outcomes

Table 4 presents the clinical outcomes between the two treatment groups. Clinical cure rate was significantly higher in the higher loading dose group (31/40 (77.5%) vs. 22/40 (55.0%), χ² = 4.48, df = 1, p = 0.034, Cramér's V = 0.24). Median time to clinical response was shorter in the higher dose group (4 vs. 6 days, p = 0.018). Duration of mechanical ventilation was significantly shorter (median = 12 vs. 16 days, p = 0.042), as was PICU length of stay (median = 18 vs. 24 days, p = 0.038). The 28-day mortality was lower in the higher-dose group (7/40 (17.5%) vs. 11/40 (27.5%)), though this difference did not reach statistical significance (p = 0.28). Figure 3 displays Kaplan-Meier curves showing the cumulative probability of clinical response over time for both treatment groups.

**Table 4:** Clinical outcomes. Abbreviations: CBA, colistin base activity; IQR, interquartile range; PICU, pediatric intensive care unit.

Outcome	Standard loading dose (5 mg/kg CBA) (n = 40)	Higher loading dose (9 mg/kg CBA) (n = 40)	p-value
Clinical cure, n (%)	22/40 (55.0)	31/40 (77.5)	χ² = 4.48, df = 1, p = 0.034, Cramér's V = 0.24
Time to clinical response (days), Median (IQR)	6 (4-9)	4 (3-7)	p = 0.018
Duration of mechanical ventilation (days), Median (IQR)	16 (10-24)	12 (8-18)	p = 0.042
PICU length of stay (days), Median (IQR)	24 (16-34)	18 (12-28)	p = 0.038
28-day mortality, n (%)	11/40 (27.5)	7/40 (17.5)	p = 0.28

**Figure 3:**
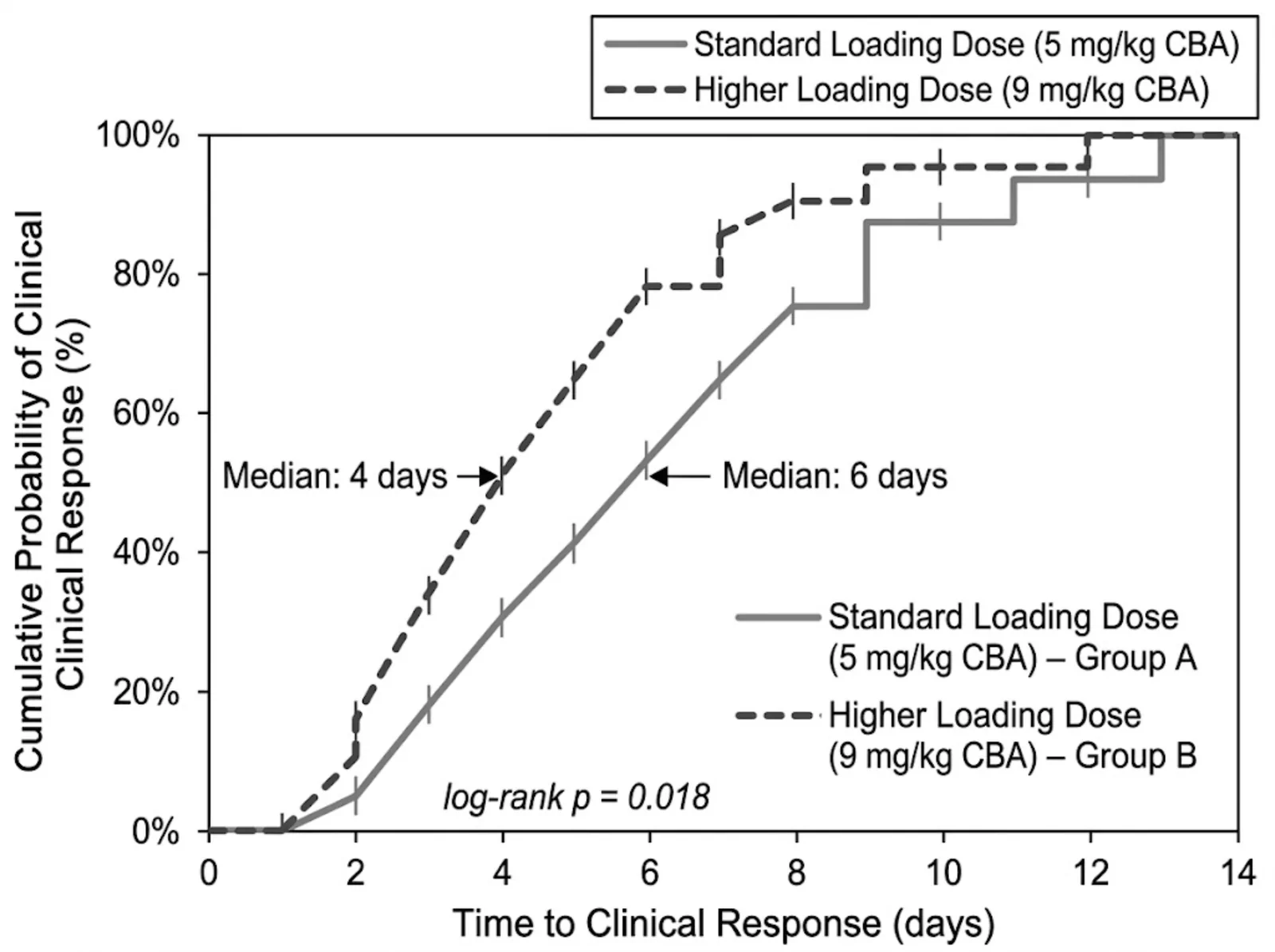
Kaplan-Meier curves for time to clinical response. The higher loading dose group (9 mg/kg CBA) achieved faster clinical response compared to the standard loading dose group (5 mg/kg CBA), with a median time to response of 4 days versus 6 days (log-rank p=0.018). Censored patients are denoted by tick marks on the curves. * indicates statistical significance (p < 0.05). Abbreviations: CBA, colistin base activity.

Colistin trough levels

Table 5 shows the colistin trough levels and the percentage of patients who reached therapeutic levels (≥2 µg/mL) on days 2 and 5. Colistin trough levels on day 2 were significantly higher in the higher loading dose group (2.8 ± 0.9 vs. 1.6 ± 0.6 µg/mL, p < 0.001). The proportion of patients achieving therapeutic trough levels (≥2 µg/mL) was significantly higher in the higher dose group (29/40 (72.5%) vs. 14/40 (35.0%), p = 0.001). Colistin trough levels were not available for eight patients (four in each group) due to logistical reasons; these patients were excluded from trough-level analyses only. Figure 4 displays colistin trough levels by day.

**Table 5:** Colistin trough levels. Abbreviations: CBA, colistin base activity; SD, standard deviation.

Parameter	Standard loading dose (5 mg/kg CBA) (n = 40)	Higher loading dose (9 mg/kg CBA) (n = 40)	p-value
Day 2 trough level (µg/mL), Mean ± SD	1.6 ± 0.6	2.8 ± 0.9	p < 0.001
Therapeutic level (≥2 µg/mL) on day 2, n (%)	14/40 (35.0)	29/40 (72.5)	p = 0.001
Day 5 trough level (µg/mL), Mean ± SD	2.2 ± 0.8	3.1 ± 1.0	p < 0.001

**Figure 4:**
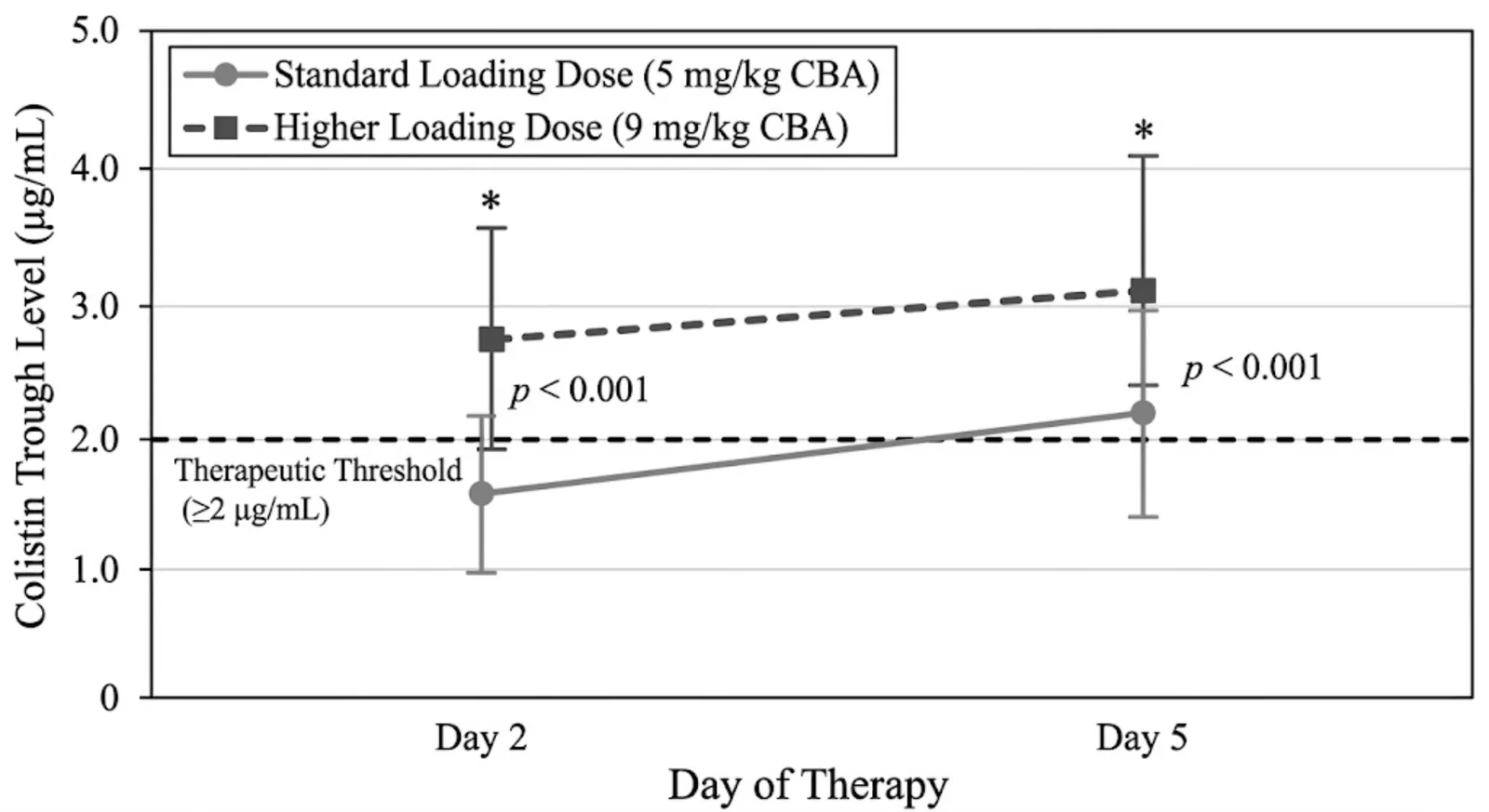
Colistin trough levels by day. This line graph shows colistin trough levels on day 2 and day 5 of therapy for both treatment groups. The higher loading dose group (9 mg/kg CBA) achieved significantly higher trough levels at both time points compared to the standard loading dose group (5 mg/kg CBA) (day 2: 2.8 ± 0.9 vs. 1.6 ± 0.6 µg/mL, p < 0.001; day 5: 3.1 ± 1.0 vs. 2.2 ± 0.8 µg/mL, p < 0.001). Error bars represent standard deviation. The horizontal dashed line at 2.0 µg/mL represents the therapeutic threshold for colistin trough levels. * indicates statistical significance (p < 0.001). Abbreviations: CBA, colistin base activity; SD, standard deviation; µg/mL, micrograms per milliliter.

Nephrotoxicity

Table 6 presents safety outcomes and nephrotoxicity incidence in both treatment groups. The incidence of nephrotoxicity was similar between groups: nine (22.5%) in the higher dose group vs. seven (17.5%) in the standard dose group (χ² = 0.31, df = 1, p = 0.58, Cramér's V = 0.06). Nephrotoxicity was predominantly Stage 1 in both groups (standard: 6/7, higher: 7/9), with Stage 2 occurring in one patient in the standard group and two patients in the higher group. No patient required renal replacement therapy.

**Table 6:** Safety outcomes. Abbreviations: CBA, colistin base activity; SD, standard deviation; RRT, renal replacement therapy.

Outcome	Standard loading dose (5 mg/kg CBA) (n = 40)	Higher loading dose (9 mg/kg CBA) (n = 40)	p-value
Nephrotoxicity (any), n (%)	7/40 (17.5)	9/40 (22.5)	χ² = 0.31, df = 1, p = 0.58, Cramér's V = 0.06
Mean increase in creatinine (mg/dL), Mean ± SD	0.3 ± 0.2	0.4 ± 0.3	0.32
RRT required, n (%)	0/40 (0)	0/40 (0)	-

Multivariate analysis

Table 7 presents the results of multivariate logistic regression analysis identifying independent predictors of clinical cure. In multivariate logistic regression, higher loading dose was independently associated with microbiological eradication (adjusted odds ratio (aOR) = 3.24, 95% CI: 1.28-8.20, p = 0.013) and clinical cure (aOR = 2.86, 95% CI: 1.12-7.30, p = 0.028). After adjusting for combination therapy as a covariate, higher loading dose remained independently associated with clinical cure (aOR = 2.67, 95% CI: 1.04-6.85, p = 0.041).

**Table 7:** Multivariate predictors of clinical cure. This table presents the results of a multivariate logistic regression analysis identifying independent predictors of clinical cure. Abbreviations: OR, odds ratio; CI, confidence interval; CBA, colistin base activity; PRISM III, Pediatric Risk of Mortality III.

Predictor	Adjusted OR	95% CI	p-value
Higher loading dose (9 vs. 5 mg/kg CBA)	2.86	1.12-7.30	0.028
PRISM III score <20	2.45	1.08-5.56	0.032
Colistin trough ≥2 µg/mL (day 2)	3.12	1.32-7.38	0.010

## Discussion

This retrospective comparative study of 80 critically ill children with VAP caused by carbapenem-resistant organisms demonstrates that a higher loading dose of colistin (9 mg/kg CBA) was associated with significantly improved microbiological eradication (33/40 (82.5%) vs. 24/40 (60.0%), p = 0.028), higher clinical cure rates (31/40 (77.5%) vs. 22/40 (55.0%), p = 0.034), faster clinical response (median 4 vs. 6 days, p = 0.018), and shorter duration of mechanical ventilation (median = 12 vs. 16 days, p = 0.042) and PICU stay (median 18 vs. 24 days, p = 0.038), while nephrotoxicity did not differ significantly between groups (9/40 (22.5%) vs. 7/40 (17.5%), p = 0.58). On multivariate analysis, higher loading dose was independently associated with microbiological eradication (aOR = 3.24, 95% CI: 1.28-8.20, p = 0.013) and clinical cure (aOR = 2.86, 95% CI: 1.12-7.30, p = 0.028), even after adjusting for combination therapy as a covariate (aOR = 2.67, 95% CI: 1.04-6.85, p = 0.041).

The improved microbiological eradication rate observed with the higher loading dose (33/40 (82.5%) vs. 24/40 (60.0%)) is clinically meaningful. This is consistent with the concentration-dependent killing of colistin and the importance of achieving rapid therapeutic concentrations [11,12]. The higher day 2 trough levels in the higher dose group (2.8 vs. 1.6 µg/mL) and the higher proportion achieving therapeutic levels (29/40 (72.5%) vs. 14/40 (35.0%)) support this mechanism.

The clinical benefits observed with the higher loading dose have important implications for patient outcomes and healthcare resource utilization. Each additional day on mechanical ventilation is associated with increased risk of VAP recurrence and PICU-acquired complications [1,3]. The four-day reduction in PICU stay in the higher dose group could translate to significant cost savings and improved bed availability. While we did not perform a formal cost analysis, the estimated reduction of approximately 1,200 Jordanian dinars (JOD) per day in PICU bed costs suggests potential cost savings of approximately 4,800 JOD per patient (192,000 JOD for the 40 patients in the higher dose group).

The lack of significant difference in nephrotoxicity between groups (9/40 (22.5%) vs. 7/40 (17.5%), p = 0.58) is reassuring, though this should not be interpreted as proof of equal safety given the limited sample size and the study's design as a superiority comparison rather than an equivalence or non-inferiority analysis [15]. This aligns with recent studies suggesting that higher loading doses of colistin may not increase nephrotoxicity when administered with appropriate monitoring [16,17,19]. Importantly, no patient in either group required renal replacement therapy, suggesting that both dosing strategies were within acceptable safety limits.

Comparison with previous studies

Our findings are consistent with the growing body of literature supporting higher colistin loading doses. Elefritz et al. (2017) reported improved clinical outcomes with a loading dose of 9 mg/kg CBA in critically ill adults [16]. Spapen et al. (2019) demonstrated that high-dose colistin was associated with improved outcomes in VAP [17]. Lu et al. (2012) found that high-dose colistin achieved better clinical outcomes in patients with MDR Gram-negative pneumonia [19]. The relationship between age, baseline kidney function, and colistin nephrotoxicity has been highlighted by Prasannan et al. (2021), who emphasized the importance of renal function monitoring [10].

Our results extend these findings to the pediatric population and provide specific data on the comparative effectiveness of two distinct loading dose strategies. Antachopoulos et al. (2021) demonstrated that higher than recommended doses of colistin were associated with improved clinical outcomes in critically ill pediatric patients, with population pharmacokinetic analysis supporting the safety and efficacy of this approach [18]. Fatehi et al. (2019) demonstrated significantly higher overall efficacy with a colistin loading dose in critically ill children [20]. Garonzik et al. (2011) showed that a colistin loading dose of 4 mg/kg CBA resulted in improved colistin exposure without increased acute kidney injury [21].

Limitations

Several limitations should be considered when interpreting our findings.

Confounding by Indication

The retrospective design introduces the possibility of selection bias. While the two dosing groups were well-balanced on measured confounders, unmeasured variables may have influenced treatment selection, clinical outcomes, or both. The dosing strategy may have been influenced by clinical factors such as perceived disease severity, underlying renal function, or clinician preference. We attempted to minimize this through multivariate adjustment for PRISM III score, organism type, and combination therapy; however, residual confounding cannot be excluded.

Temporal Bias

The non-randomized comparison of two distinct loading dose strategies may have been influenced by temporal changes in clinical practice, pathogen epidemiology, or institutional protocols during the three-year study period. The protocol change from 5 mg/kg to 9 mg/kg CBA occurred in January 2024, and changes in clinical care or pathogen susceptibility patterns during this period may have influenced outcomes independently of the dosing strategy.

Single-Center Design

The single-center design may limit generalizability to other healthcare settings with different patient populations, clinical practices, or pathogen epidemiology.

Small Sample Size

The sample size (n = 80) limited power for subgroup analyses and safety comparisons. The nonsignificant difference in nephrotoxicity (22.5% vs. 17.5%, p = 0.58) does not demonstrate equivalence or confirm that the higher dose is equally safe. Confidence intervals for the safety comparison are wide and should be interpreted cautiously.

Misclassification of VAP and Microbiological Eradication

Our definition of eradication included a reduction in colony count rather than only a negative culture, which may introduce subjectivity and misclassification bias. The diagnosis of VAP based on clinical and microbiological criteria may also be subject to misclassification.

Exclusion of Patients Receiving Other Nephrotoxic Drugs

The exclusion of patients receiving other nephrotoxic agents may underestimate nephrotoxicity in our cohort and limit generalizability to typical PICU practice where combination nephrotoxic therapy is common.

Missing Trough-Level Data

Colistin trough levels were not available for eight patients (four in each group) due to logistical reasons, which may introduce bias in the pharmacokinetic analyses.

Multiple Comparisons

Multiple secondary comparisons were performed without adjustment for multiplicity, increasing the possibility of type I error. Results for secondary outcomes should therefore be interpreted as exploratory rather than confirmatory.

Regression Model Limitations

The number of outcome events relative to the number of potential covariates in the regression models may limit the stability of the estimates. The small sample size precluded inclusion of all potentially relevant covariates.

Absence of Long-Term Follow-Up

Longer-term outcomes beyond PICU discharge were not assessed, and the impact on long-term renal function and survival remains unknown.

Given these limitations, our findings demonstrate associations between the higher loading dose and improved outcomes but do not establish causation. The absence of statistical significance for toxicity should not be interpreted as proof of equal safety. These findings are hypothesis-generating and require confirmation in larger, prospective, preferably multicenter randomized studies.

Future research directions

Future prospective, randomized controlled trials are needed to confirm these findings and establish optimal dosing strategies in this vulnerable population. Such studies should include larger sample sizes with adequate power for safety comparisons, standardized protocols for colistin administration and therapeutic drug monitoring, comprehensive assessment of renal function using standardized criteria, long-term follow-up to assess durability of clinical benefits and late renal effects, and formal cost-effectiveness analyses to guide healthcare resource allocation. Additionally, pharmacokinetic studies in specific pediatric subpopulations (e.g., infants, older children, and obese patients) would help refine dosing recommendations.

## Conclusions

A higher loading dose of colistin (9 mg/kg CBA) in children with VAP caused by carbapenem-resistant organisms was associated with improved microbiological eradication, higher clinical cure rates, faster clinical response, and shorter duration of mechanical ventilation and PICU stay, while nephrotoxicity did not differ significantly between the two dosing groups. However, given the retrospective observational design, these results support an association rather than causation, and the nonsignificant difference in nephrotoxicity should not be interpreted as proof of equal safety. These findings are hypothesis-generating and require confirmation in larger, prospective, preferably multicenter randomized studies. Clinicians should consider the higher loading dose as a potential strategy for achieving rapid therapeutic concentrations in critically ill children with MDR Gram-negative VAP, while maintaining vigilant monitoring for nephrotoxicity and individualizing therapy based on renal function and clinical response.
